# Ecological analysis of social risk factors for Rotavirus infections in Berlin, Germany, 2007–2009

**DOI:** 10.1186/1476-072X-11-37

**Published:** 2012-08-28

**Authors:** Hendrik Wilking, Michael Höhle, Edward Velasco, Marlen Suckau, Tim Eckmanns

**Affiliations:** 1 Department for Infectious Disease Epidemiology, Robert Koch Institute, Berlin, Germany; 2 Senate of Berlin's Department for Health, Environment and Consumer Protection, Berlin, Germany

**Keywords:** Rotavirus infection, Urban health, Disease clustering, Social environment, Risk factors, Bayesian inference

## Abstract

**Background:**

Socioeconomic factors are increasingly recognised as related to health inequalities in Germany and are also identified as important contributing factors for an increased risk of acquiring infections. The aim of the present study was to describe in an ecological analysis the impact of different social factors on the risk of acquiring infectious diseases in an urban setting. The specific outcome of interest was the distribution of Rotavirus infections, which are a leading cause of acute gastroenteritis among infants and also a burden in the elderly in Germany. The results may help to generate more specific hypothesis for infectious disease transmission.

**Methods:**

We analysed the spatial distribution of hospitalized patients with Rotavirus infections in Berlin, Germany. The association between the small area incidence and different socio-demographic and economic variables was investigated in order to identify spatial relations and risk factors. Our spatial analysis included 447 neighbourhood areas of similar population size in the city of Berlin. We included all laboratory-confirmed cases of patients hospitalized due to Rotavirus infections and notified between 01/01/2007 and 31/12/2009. We excluded travel-associated and nosocomial infections. A spatial Bayesian Poisson regression model was used for the statistical analysis of incidences at neighbourhood level in relation to socio-demographic variables.

**Results:**

Altogether, 2,370 patients fulfilled the case definition. The disease mapping indicates a number of urban quarters to be highly affected by the disease. In the multivariable spatial regression model, two risk factors were identified for infants (<4 year olds): Rotavirus incidence increased by 4.95% for each additional percent of unemployed inhabitants in the neighbourhood (95% credibility interval (CI): 3.10%-6.74%) and by 0.53% for each additional percent of children attending day care in the neighbourhood (95% CI: 0.00%-1.06%). We found no evidence for an association with the proportion of foreign residents, population density, the residential quality of accommodations and resident changes in the neighbourhood.

**Conclusions:**

Neighbourhoods with a high unemployment rate and high day care attendance rate appear to be particularly affected by Rotavirus in the population of Berlin. Public health promotion programs should be developed for the affected areas. Due to the ecological study-design, risk pathways on an individual patient level remain to be elucidated.

## Background

### Rotavirus epidemiology

Rotavirus infections can be repeatedly acquired in persons from birth to old age, although infants in their first years are mostly affected [1]. The virus is highly infectious, and it can be assumed that after one year about 2/3 of the children have experienced at least one Rotavirus infection and 1/3 a second Rotavirus infection. After two years almost all children have undergone a Rotavirus infection, 2/3 a second and 1/3 a third Rotavirus infection [2]. The appearance of most of these infections is either asymptomatic or an episode of mild enteric symptoms, which an infected person may not notice. Despite this, a large number of infections lead to severe disease courses with diarrhoea, need for hospitalization and, in rare cases death [3]. There are considerable differences, however, in disease severity between primary and subsequent infections [2].

On a population-level Rotavirus infections are the leading cause of acute gastroenteritis among infants and young children in Germany [4], in Europe [5] and worldwide [6]. The global disease burden of Rotavirus infections for the health care systems can be regarded as high in developing countries, as well as in countries with more advanced economies. The overall worldwide annual mortality of Rotavirus infection is estimated approximately 440,000 deaths, mostly in infants. Rotavirus associated deaths occur almost exclusively in developing countries [7].

Family structures and social facilities such as day care centres for infants as well as nursing homes for the elderly can facilitate the transmission of the virus, and repeatedly lead to Rotavirus outbreaks. The community spread also seems to be influenced by the climate, leading to a characteristic seasonality with peaks in spring-time [8,9]. In addition, nosocomial Rotavirus infections are also frequent [10].

In 2006, two Rotavirus vaccines (Rotarix and RotaTeq) were licensed and introduced in Germany [11,12].Although currently under consideration by the German Standing Committee on Vaccination, the vaccines are not yet integrated in the routine childhood immunization schedule in Germany. Given the potential for a change in community spread due to expected differences in vaccine coverage in the population, there may thus be a prevalence of groups at special risk that are not currently in focus, but should be. Further understanding of risk factors for Rotavirus distribution on a population level is therefore needed in order to guide future decisions.

### Social environments and health outcomes

The importance of links between the social environment and infectious diseases has long been of focus in the developing world, since these regions are burdened with issues that increase the burden of infectious diseases, such as poverty and suboptimal living conditions. Although less pronounced, issues like income inequality are important and relevant in Europe as well as in other advanced economic regions [13]. A part of these groups contain individuals from a marginalized and segregated subpopulation that may not be fully integrated into the general population. In Germany, where poverty and poor housing conditions are also important issues, health inequalities are on the rise due to changes in, e.g., economy and migration [14,15]. Recent position papers have urged the importance of identifying vulnerable groups on an ecological level [16] complementing strictly individual-based epidemiology on risk factors with population-based approaches, where socioeconomic factors are linked to health outcomes on a spatial aggregate level becomes important [17,18].

Due to its eventful history Berlin is a city with highly diverse communities in distinctive neighbourhoods, which makes it an ideal prototype to study these influences on a spatial level. In the future these neighbourhoods could form a new level of surveillance, mitigation and containment of infectious diseases in Berlin and elsewhere.

While people often seek out their place of residence based on their social and financial (socioeconomic) status, the social characteristics in those places of residence might reflexively influence people’s social status [19]. These two forces – seeking out and being influenced by – socioeconomic status might contribute to the formation of distinct living environments, which reflect the social diversity of societies especially in metropolitan areas. This can have an impact on the transmission of infectious diseases, which was shown for North American cities and which might also be true for European cities [20].

### Spatial regression analysis of surveillance data

There has been some recent work in the literature that uses mathematical modeling to describe the dynamics of rotavirus at the population level [21]. Models are calibrated using time series data at the national or state level. Since the effect of socio-demographic variables is expected to happen at a much more detailed spatial scale, a more spatial oriented analysis approach is needed for such investigation. However, a good spatial resolution of health data is often in conflict with data privacy issues and small scale spatial analysis of infectious disease data is accordingly underdeveloped in practice. Another hindrance is that advanced statistical methodology is needed in order to properly take the spatial nature of such data into account: if one falsely relies on the common statistical assumption of observations being independent and identically distributed, one is overrating the value of information in the data and might obtain wrong conclusions in statistical significance tests. Thus, statistical methodology and corresponding software implementations should go beyond the independence assumption for such data. Spatial Bayesian regression models are the most common analysis tool in such situations [22]. These models have been successfully applied, e.g. for cancer incidence data [23], mortality data [24], but also for worldwide malaria incidence [25], nationwide surveillance data on Shiga toxin-producing *Escherichia coli* in Germany [26] and tuberculosis in urban setting in Brazil [27]. Recently, the integrated nested Laplace approximation (INLA) approach together with its implementation has provided a new and efficient method to perform statistical inference for spatial regression models including Gaussian random effects [28]. For example, the method has already been successfully applied for infectious disease surveillance data in veterinary medicine in Switzerland [29,30]. We employ this technique for the analysis of Rotavirus incidence – see methods section for details. Thus, our analysis covers all aspects of small area infectious disease epidemiology while simultaneously employing advanced statistical methods for the analysis.

### Objective

The objective of this study was to describe the small-area spatial distribution of the incidence of hospitalized Rotavirus cases in Berlin. A further aim was to explain parts of the spatial distribution pattern using socio-economic and socio-demographic variables.

## Results

### Descriptive analysis

More than one third (36.9%) of the analysed hospitalized Rotavirus cases were in the age group <1 year with an average yearly incidence of 969 in 100,000 population. There was a decreasing trend of incidence with increased age until adulthood with 2 cases in 100,000 (Table 1). This trend was reversed in the elderly (≥60 years) with an incidence of 14 cases in 100,000. The age group of ≥60 years shows the longest median duration of hospitalization. However, the size of the Interquartile range (IQR) as well as application of a Kruskal–Wallis rank test (p = 0.07) indicates that there is only weak evidence for differences between the lengths of hospitalization between the age groups. Overall, the sex distribution of cases was almost even. Furthermore, 15.3% of the hospitalized cases were reported as part of a larger outbreak, although there were large differences between age groups. Altogether, one fatal outcome in an infant less than one year old was reported.

**Table 1:** Age distribution of hospitalized Rotavirus cases in Berlin 2007-2009

**Age group**	**Number of cases (percent of all cases)**	**Average yearly incidence [95% CI**^**a**^**] (in 100,000)**	**Median length of hospitalization [IQR**^**b**^**] (days)**	**Male gender (%)**	**Part of an outbreak (%)**
<1 year	872 (36.9)	969 [862–1,087]	4 [2–5]	53.7	13.1
1 - <2 years	639 (27.0)	668 [582–763]	3 [2–4]	55.6	15.2
2 - <3 years	204 (8.6)	219 [170–278]	3 [2–4]	56.9	20.6
3 - <4 years	72 (3.0)	82 [53–122]	3 [2–4]	44.6	19.4
4 - <6 years	73 (3.0)	42 [27–63]	3 [2–4]	50.6	21.9
6 - < 20 years	64 (2.7)	6 [3–9]	3 [2–5]	54.7	9.4
20 - < 60 years	98 (4.1)	2 [1–2]	4 [2–5]	50.0	6.1
≥60 years	348 (14.7)	14 [12–17]	5 [4–7]	33.9	18.7
total	2,370 (100)	23 [22–25]	4 [2–5]	51.1	15.3

^a^ = confidence interval; ^b^ = Interquartile range.

### Disease mapping

**Figure 1:**
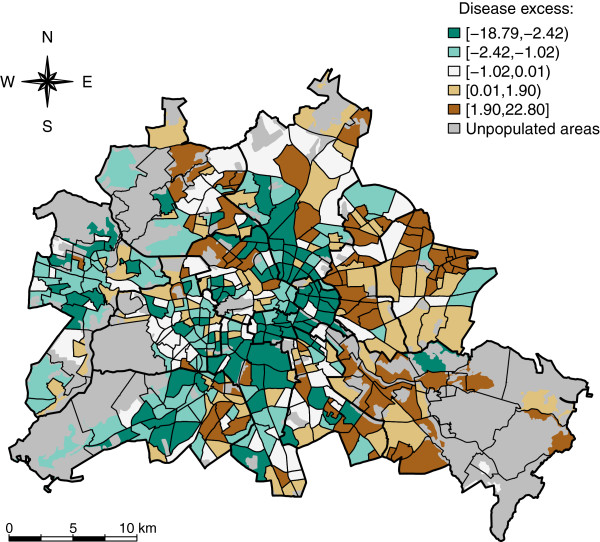
**Spatial distribution of hospitalized cases of Rotavirus infections in 447 neighbourhoods in Berlin.** Age- standardized absolute disease excess in quintiles.

Visually, the age standardized absolute excess of hospitalized Rotavirus cases in Berlin were spatially unevenly distributed and show some degree of spatial association (Figure 1). There was a concentration of neighbourhoods with high values in the southeast and most parts of the central east and northeast. Here, most spatial units were in the two upper quintiles. Some neighbourhoods in the northwest and southwest also show high disease burden as well as a much more heterogeneous distribution. Most neighbourhoods in the central parts of Berlin were in the lowest quintile.

### Regression analyses

Regression analysis were performed separately for infant (<4 years) and elderly (≥60 years) as these are the two age groups particularly at risk. The results of the univariable spatial regression analyses of the infant model (<4 years) showed that, as expected, the age category-variable exhibits the by far lowest excess risk ratios (Table 2). Ecological variables where the credibility intervals for the relative risk did not contain the one were: unemployment and day care attendance rate. Low influence was detected for the variables migration volume, population density, residential quality and foreign residents. This multivariable model identified two risks for the occurrence of hospitalized Rotavirus cases: incidence increased by 4.95% for each percent increase in unemployed inhabitants in the neighbourhood (95% credibility interval (CI): 3.10%-6.74%). Furthermore, incidence increased by 0.53% for each percent increase of children attending day care (95% CI: 0.00%-1.06%). The other variables in the multivariable model had effects where the credibility intervals for the relative risk contained the one, i.e. given the available data the influence of these variables cannot be distinguished from chance effects when adjusting for all other variables.

**Table 2:** Estimation results of univariable and multivariable analysis (age group from <1 to <4 years)

**Explanatory variable**	**Univariable analysis**		**Multivariable analysis**	
	**Excess risk ratio (95% CI)**	**DIC (rank)**	**Excess risk ratio (95% CI)**	**DIC**
Unemployment^a^	3.94 (2.37, 5.49)	3623.9 (1)	4.95 (3.10, 6.74)	3627.4
Migration volume^a^	0.56 (−0.30, 1.42)	3637.2 (4)	−0.04 (−1.01, 0.93)	
Foreign residents^a^	0.62 (−0.31, 1.56)	3635.8 (2)	−0.25 (−1.36, 0.88)	
Population density^a^	0.04 (−0.04, 0.12)	3636.4 (3)	−0.00 (−0.09, 0.08)	
Basic residential quality^a^	−0.02 (−0.20, 0.15)	3638.7 (7)	−0.14 (−0.32, 0.04)	
Day care attendance^b^	0.25 (0.26, 0.77)	3637.6 (6)	0.53 (0.00, 1.06)	
<1 year^a^	reference^c^	3637.3 (5)	reference^d^	
1 - <2 years^a^	−30.89 (−37.72, -23.60)		−43.74 (−56.04, -29.19)	
2 - <3 years^a^	−77.34 (−80.64, -73.74)		−84.10 (−89.65, -76.66)	
3 - <4 years^a^	−91.60 (−93.49, -89.46)		−94.50 (−96.84, -91.12)	

Fixed-effect of explanatory variables on hospitalized Rotavirus infections (infant model) in Berlin. Excess risk ratio = (Relative risk-1)·100%; 95% CI: 95% Bayesian credibility interval, ^a^ = in percent, ^b^in percent stratified for the four age groups ^c^To assess the relevance of the excess risk ratios: the baseline three-year-incidence in the model with just age group as covariate is estimated to be 266/10,000 inhabitants (i.e. posterior mean of the age group of <1 year olds), ^d^Posterior mean three-year-incidence of the age group of <1 year olds is 189/10,0000 inhabitants.

The results for the univariable and multivariable regression in elderly (≥60 years) are shown in Table 3. The only confirmed risk factor was population density of the neighbourhoods. In contrast the proportion of infants (<4 years) in the population of the neighbourhoods was not a risk factor for hospitalized Rotavirus infections in the elderly. However, it has to be noted that for the elderly, 64% of the neighbourhoods have zero cases, 21% have one case and the remaining have up to a maximum of 19 cases. As a consequence, results are more variable than in the infant model.

**Table 3:** Estimation results of univariable and multivariable analysis (age group from 60 years and above)

**Explanatory variable**	**Univariable analysis**		**Multivariable analysis**	
	**Excess risk ratio (95% CI)**	**DIC (rank)**	**Excess risk ratio (95% CI)**	**DIC**
Infants^a^	0.87 (−15.15, 18.70)	844.58 (6)	−2.56 (−20.30, 17.69)	845.8
Unemployment^a^	2.00 (−2.65, 6.78)	843.65 (3)	4.59 (−1.18, 10.56)	
Migration volume^a^	1.12 (−1.13, 3.29)	844.10 (4)	1.86 (−0.93, 4.45)	
Foreign residents^a^	−0.95 (−3.45, -1.57)	845.24 (7)	−1.83 (−4.92, 1.27)	
Population density^a^	−0.25 (−0.49, -0.01)	842.92 (1)	−0.30 (−0.56, -0.04)	
Basic residential quality^a^	0.02 (−0.42, 0.45)	844.51 (5)	−0.01 (−0.49, 0.46)	

Fixed effects of explanatory variables for hospitalized Rotavirus infections (elderly model) in Berlin. Excess risk ratio = (Relative risk-1)·100%; 95% CI: 95% Bayesian credibility interval, ^a^in percent.

### Analysis of spatial effects

**Figure 2:**
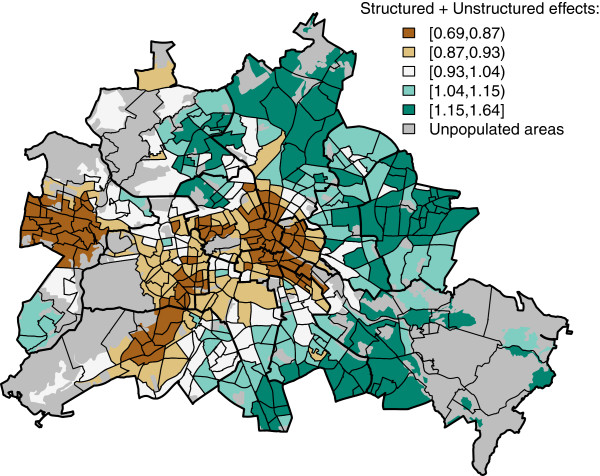
**Relative risk due to combined structured spatial effects *ψ***_***i ***_**and unstructured spatial heterogeneity *υ***_***i ***_**from the final multivariable regression model in 447 neighbourhoods in Berlin, Germany.** In quintiles.

**Figure 3:**
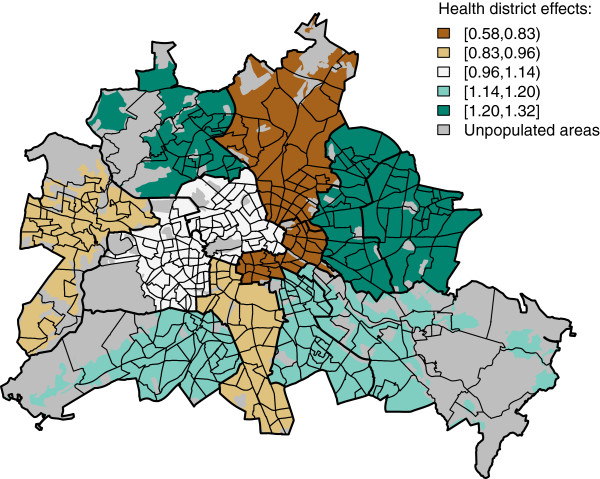
**Relative risk due to unstructured spatial heterogeneity *α***_***j(i) ***_**in the final multivariable regression model in 12 health districts in Berlin, Germany.**

The plotting of the spatial effects of the neighbourhoods reveals residual variation in the relative risk after accounting for measured covariates. Figure 2 shows the combination of structured and unstructured spatial effects on a relative risk scale, i.e. exp(*ψ*_*i+*_*υ*_*i*_). Neighbourhoods with negative residual values are in central and western parts of Berlin. The areas with negative residual values cover a crescent-shaped area around the city centre to the north, east and south. These could be caused by missing explanatory variables, which exhibit their own spatial structure, i.e. mean education level in the neighbourhood or can be caused by endogenous effects on the incidence of hospitalized Rotavirus cases within the city of Berlin, e.g. due to different diagnostic procedures in hospitals. Alternatively this reflects regional differences in disease prevalence (i.e.: small pockets of exceptional higher and lower vaccination coverage, respectively). A plot of the unstructured spatial heterogeneity due to the 12 health districts, i.e. exp(*α*_*j(i)*_), in the infant model is shown in Figure 3. The results can be explained by health-district specific reporting artefacts or different regional awareness.

## Discussion

### Key findings

We identified associations between socioeconomic factors as risk factors and the incidence of severe Rotavirus infections leading to hospitalization in an urban setting in Germany. In addition, it is the first time that socio-demographic and socio-economic factors are analysed to explain variations of infectious diseases in Berlin at such a detailed spatial scale. This study suggests that socioeconomic risk factors may affect the distribution of infectious diseases in Berlin, and that spatially detailed information about infectious disease cases can provide more specific information about these effects.

Both the occurrence and perhaps the severity of disease could also be explained by spatial variations in vaccination coverage. WHO recommends that a surveillance system for severe Rotavirus infections should be in place to monitor the effect of universal childhood rotavirus vaccination once implemented [31]. The German surveillance system is recognized as being able to monitor the impact of the vaccines [4]. The expected changes in burden of disease could be monitored more specifically if information on socioeconomic determinants for specific areas and subgroups would be assignable and accessible for analysis. Unfortunately, data on vaccination coverage with the required spatial precision as in our study is not available. In our study, unemployment is identified as a strong risk factor for disease. The investigation of the underlying causality of this socioeconomic factor requires the setup of appropriate analytical strategies involving multiple interrelated variables [17,18]. These include proximal biologic precursors (e.g. nutrition) and more distal and contextual risk factors like behaviour or unemployment as psychological burden. Unemployment in families is related to child poverty and maybe connected to poor access to medical care (i.e. Rotavirus vaccination is not generally covered by health insurance programs). Alternatively, unemployment could act as a cofactor for educational background or different risk behaviour regarding hygiene and oral rehydration.

### Limitations and strength

The interpretation of the results is limited by the ecological design of the study. The statistical association of unemployment can be explained by the influence of the social situation of the neighbourhoods on the frequency and severity of the disease in all children in the neighbourhoods with high levels of unemployment. This would be a true ecological effect. Alternatively, the effect on the frequency and severity of the disease was observed in the underprivileged sub-group only. Furthermore, it is conceivable that the likelihood of hospitalization is increased in children of families burdened by unemployment, due to differential access to hospital services or, in cases of gastroenteritis, to different routines for referral by general practitioners. Although an analysis with non-hospitalized Rotavirus cases included shows similar associations (data not shown). Similarly, the association of day care attendance rate can be explained as an increased risk in *all* children attending day care of the respective neighbourhood or only fraction attending day care in the respective neighbourhood.

Altogether, the issue of bias remains a major limitation of any ecological analysis such as our [32]. As a consequence, the results of our analysis should be interpreted with care – not only because of possible ecological bias, but also due to possible confounding due to location, i.e. changes in covariate effects due to the addition of a location based structured random variable in the model [33].

However, despite its limitations ecological analyses remain an important hypothesis generating tool where already readily available registry data can be used to provide first answers to questions of public health importance. A confirmation of the results by an individual-level based analyses, e.g. through a case–control study, would be the natural next step.

Reporting of laboratory-notified Rotavirus infections in Berlin is mandatory with continuous case definition; however, eligibility for testing is not defined and therefore case ascertainment could vary inside the study area. Under the German reimbursement system for hospitalization charges, hospitals receive higher payments for cases of acute gastrointestinal enteritis with a confirmed infectious agent and therefore have a high incentive for the commission of Rotavirus diagnostic. Further steps in quantifying possible consequences of reporting artefacts could be to perform a joint modelling together with another gastrointestinal disease such as, e.g. Norovirus, as done in Held et al. [34]. However, we believe that our analysis restricted to hospitalized cases only provides results that reduce ascertainment bias.

The results of our study emphasize the relevance of spatial regression models including spatially structured effects and unstructured spatial heterogeneity for the detection of risk factors.

## Conclusions

Appropriate targeting of public health promotion programs could be achieved by combining the criteria of neighbourhood with the risk groups identified in this study. The influence of the day care attendance rate indicates that high requirements for hygiene in child day-care facilities should be regularly checked and routinely maintained. Due to different policies in Germany, the attendance rates for day care centres of infants are unequal. Since 2008, a federal law supports the expansion of day care centres, and realizes the legal claim of all parents throughout Germany to a place their child in a day care centre in 2013 [35]. Thus, this difference is of future relevance for public health services in regions in West-Berlin and Western Germany with expected increase of children attending day care in the next years.

Subsequent small-area studies of Rotavirus or other infectious diseases could provide further insight into spatial effects on disease risk. Individual based risk factors have to be refined and integrated in the future.

## Methods

### Case data

**Figure 4:**
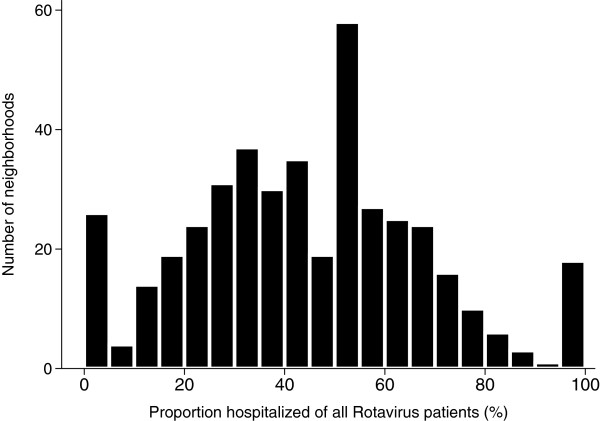
**Distribution of the proportion of hospitalized Rotavirus infections to all notified Rotavirus infection in 447 neighbourhoods Berlin, Germany.** Median: 26.3% and IQR: 43.8%-57.9%.

Rotavirus cases were notified by laboratories to the 12 local public health offices in Berlin, anonymised and transmitted via the State Office of Health and Social Affairs, Berlin, to the Robert Koch Institute [36]. We restricted our analysis to patients who were hospitalized in order to avoid case ascertainment bias, since eligibility for a laboratory diagnostic test is unclear in outpatients and may have led to differences in notification rates in Berlin neighbourhoods (Figure 4). The case definition included all clinical and laboratory-confirmed patients with Rotavirus infection, which were hospitalized due to Rotavirus between 01 January 2007 and 31 December 2009 with residence in Berlin (n = 3270). Patients having no date or a date of hospitalization prior to the date of onset of illness were excluded as definite or probable nosocomial infections (n = 846). Furthermore, we excluded 14 travel-related patients as well as 40 with unknown residence. All in all, 2370 hospitalized Rotavirus cases were analysed in this study. Transmitted data included information on age, sex, laboratory results, onset of illness, start and end date of hospitalization.

### Geo-referencing and disease mapping

Case location is given in a geographical reference system used for the urban infrastructure in Berlin [37,38]. This consists of 447 spatial units with a median population of 6631 inhabitants per unit (Interquartile range (IQR): 3960 – 9974). We refer to these units as neighbourhoods. Descriptions of the polygons for mapping were obtained from the Statistical Bureau Berlin-Brandenburg. The Rotavirus cases were geo-referenced to their neighbourhoods at the local health departments according to their home addresses. We estimated the expected number of cases *e*_*i*_ in each neighbourhood *i* that would have been observed if the incidence of disease had had the same age structure as the incidence in the whole study area:

$$\begin{array}{c} e_{i}=\sum_{a}\frac{\sum_{i}y_{i,a}}{\sum_{i}n_{i,a}}\cdot n_{i,a}, \end{array}i=1,\ldots ,447\text{,}$$

where *y*_*i,a*_ is the observed number of cases of the disease in the age strata *a* of the i’th neighbourhood and *n*_*i,a*_ is the number of residents in the strata *a* of the *i*’th neighbourhood. In our case, *a* denotes the 13 age groups (<1 year, 1- < 2 years, 2- < 3 years, 3- < 4 years, 4- < 6 years, 6- < 10 years, 10- < 20 years, 20- < 30 years, 30- < 40 years, 40- < 50 years, 50- < 60 years, 60- < 70 years, ≥70 years). For the comparison of expected and observed counts we produced values for the *absolute disease excess* (*z*_*i*_) in a neighbourhood *i* as *z*_*i*_ *= y*_*i*_*-e*_*i*_, see Figure 1. We choose to illustrate absolute excess in this descriptive figure, since absolute excess immediately contains information about the relevance of the differences (as opposite to relative risk), which is favourable when communicating results on a purely descriptive basis. Note that the subsequent modelling happens on a relative risk scale.

### Spatial Bayesian Poisson regression model

**Figure 5:**
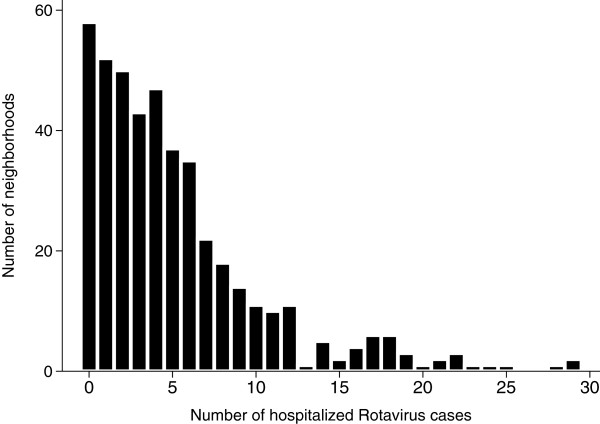
Distribution of hospitalized Rotavirus infections in the 447 neighbourhoods in Berlin, Germany.

When mapping the incidence of a rare and non-infectious disease, a common model is the spatial Poisson model, where the number of disease cases per spatial unit is assumed to follow a Poisson distribution with expectation *λ*_*i*_. The unadjusted number of hospitalized Rotavirus cases in our study, however, exhibited extra-Poisson distribution and had an asymmetrical statistical distribution (Figure 5). One of the explanations for this effect is the infectious character of rotavirus which can cause clustering. To address this infectious disease aspect we include a spatial unstructured random effect $\psi_{i}\sim N\left(0,\sigma^{2}\right)$ to allow for extra-Poisson variation while staying within a Poisson likelihood framework. Furthermore, a spatial structured effect *υ*_*i*_ was incorporated for each of the 447 neighbourhoods using a Gaussian Markov random field in order to address spatial dependence between the neighbourhoods [39]. Finally, a hierarchical unstructured random effect was included for each of the 12 health districts that each neighbourhood *i* was located in, i.e. $\alpha_{j\left(i\right)}\sim N\left(0,\tau^{2}\right)$, which accounts for reporting artefacts at local health district level and thus also allows for a hypothesized east–west difference originating from pre-reunification times.

**Figure 6:**
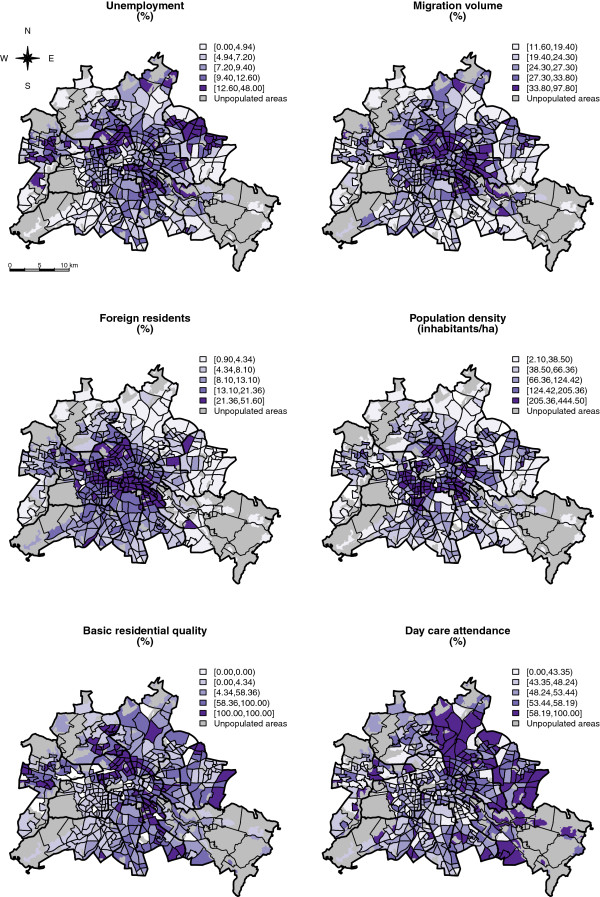
**Spatial distribution of the six covariates in the 447 neighbourhoods of Berlin.** For daycare attendance the proportion of <4 year olds which attend daycare is mapped. Note that in the regression modeling daycare attendance enters as the proportion of kids in the corresponding age group (<1, 1- < 2, 2- < 3, 3- < 4).

Independent variables include factors, which were presumably related to spatial variation of disease risk in Berlin and are incorporated as fixed effects inside the model. A full description of the explanatory variables is given in Table 4 and maps illustrating their spatial distribution in the study area are shown in Figure 6.

**Table 4:** Summary statistics of variables in 447 neighbourhoods in Berlin

**Variable**	**Median value**	**Interquartile range**	**Full definition**
Unemployment^1^	8	6-12	Proportion of unemployed persons in percent of inhabitants between 15 and 65 years of age. Source: Senate of Berlin's Department for Urban Development. The unemployment rate is highly correlated to other job market related variables. Key variable for the economic status of the inhabitants in the respective neighbourhood. Query date: 31 December, 2008
Migration volume^1^	26	21-31	Sum of all moving to and away from the neighbourhood in percent of inhabitants in the year 2008. Key variable for the dynamic and extent of environmental changes in the area (i.e. gentrification). Source: Senate of Berlin's Department for Urban Development. Query date: 31 December, 2008
Foreign residents^1^	10	6-17	Proportion of foreign residents to all inhabitants. Source: Senate of Berlin's Department for Urban Development. Query date: 31 December, 2008
Population density^2^	97	44-173	Population density defined as number of inhabitants per hectare settlement area of the neighbourhood. This is a possible proxy for the average frequency of social contact in the respective neighbourhood. Source: Senate of Berlin's Department for Urban Development. Query date: 31 December, 2008
Basic residential quality^1^	27	0-96	Basic residential quality defined as the proportion of the lowest residential quality class on all three quality classes of the Berlin rent index in the year 2007. Source: Senate of Berlin's Department for Health, Environment and Consumer Protection
Infants^1^	3	8-4	Proportion of inhabitants < 4 years in relation to all inhabitants in the neighbourhoods in percent.
Day care attendance (<1 year)^1^	1	0-3	Proportion of children attending day care centres to all children in the age groups: <1 year; 1 - <2 years; 2 - <3 years and 3 - <4 years. Source: Senate of Berlin's Department for Education, Science and Research. Query date: 31 December, 2009
Day care attendance (1 - <2 years)^1^	39	29-55	
Day care attendance (2 - <3 years)^1^	73	62-83	
Day care attendance (3 - <4 years)^1^	89	82-99	

^1^ percent, ^2^ number of inhabitants, ^3^ number of cases.

Altogether, the total number of cases for age group *a* in neighbourhood *i* is in our spatial regression model given by:

$$\begin{array}{c} y_{i,a}\sim Po\left(\lambda_{i,a}\right),\\ \eta_{i,a}=log\left(\lambda_{i,a}\right)=log\left(n_{i,a}\right)+\mu +\upsilon_{i}+\alpha_{j\left(i\right)}+\psi_{i}+z_{i}^{'}\beta \end{array}$$

Here, *n*_*i,a*_ denotes the population in neighbourhood *i* in age group *a* and *z*_*i*_ denotes the vector of explanatory variables which contains the age groups as factor variable. The intercept *μ* was the estimated value when all predictors were zero (continuous covariates) or at their baseline values (discrete covariates), respectively. For the Bayesian inference, we took the integrated nested Laplace approximation (INLA) approach as introduced by [28] and implemented in the R package *R-INLA*[40,41]. Altogether, our statistical approach resembles the one used before by Schrödle et al. [29,30]. Deviance information criterion (DIC) [42] was used as a measure for comparing Bayesian models, since it provides a trade-off between model fit and model complexity. Thus, we searched for the model with the lowest DIC.

Because we assumed different risk profiles for infants and elderly, the regression analysis was performed in two separate models: One for infants, i.e. the <4 year old containing four age groups (<1, 1- < 2, 2- < 3 and 3- < 4 years) and one for elderly, i.e. the ≥60-years-old containing a single age group. In the infant model the age group specific population was included as an offset variable to control for the strong effect of age in the infant model. Following the suggestions in Rothman et al. [43] we decided to start the investigation by performing a univariable spatial modelling of all potential risk factors adjusting only for the strong effect of age using the above model. The actual underlying multiple-inference question of identifying relevant risk factors while adjusting for the joint conglomerate of risk factors was addressed by fitting a single multivariable model containing all potential risk factors. Thus, we abandon any type of step-wise model selection, which is known to have low efficiency and poor power. Based on DIC, a health-district effect was only included in the infant model.

We used Bayesian inference and report the resulting excess risk ratios as point estimate (posterior mean) and 95% credibility intervals as a quantification of parameter uncertainty. Residual relative risk of the combined structured and unstructured spatial effects, i.e. the posterior median of exp*(ψ*_*i +*_*υ*_*i*_*),* and of the 12 health districts, i.e. exp*(α*_*j(i)*_*),* for the final multivariable regression model of the infants <4 years were mapped. In a sensitivity analysis (not-shown), possible non-linearity of the covariates was investigated; see Natário and Knorr-Held [44] for a discussion of the method and the consequences of ignoring non-linearity. However, our analyses showed that linear effects of the covariates were adequate for our purposes.

All computations and map visualizations were done in R v. 2.15.0 [41].

## Competing interests

The authors declare that they have no conflict of interest. This study and the authors were not sponsored by an external funding source.

## Authors’ contributions

HW designed the study, carried out the analysis and drafted the manuscript. MH carried out the analysis and wrote the description of the statistical methods. EV participated in the design of the study and helped to draft the manuscript. MS and TE conceived the study, participated in its design and coordination. All authors read and approved the final manuscript.
